# Rats, Global Poverty, and Paying the Piper

**DOI:** 10.3201/eid1310.000000

**Published:** 2007-10

**Authors:** Polyxeni Potter

**Affiliations:** * Centers for Disease Control and Prevention, Atlanta, Georgia, USA

**Keywords:** Rats, poverty and disease, Rembrandt van Rijn, etching, global poverty, humanities and science, art and science, about the cover

**Figure:**
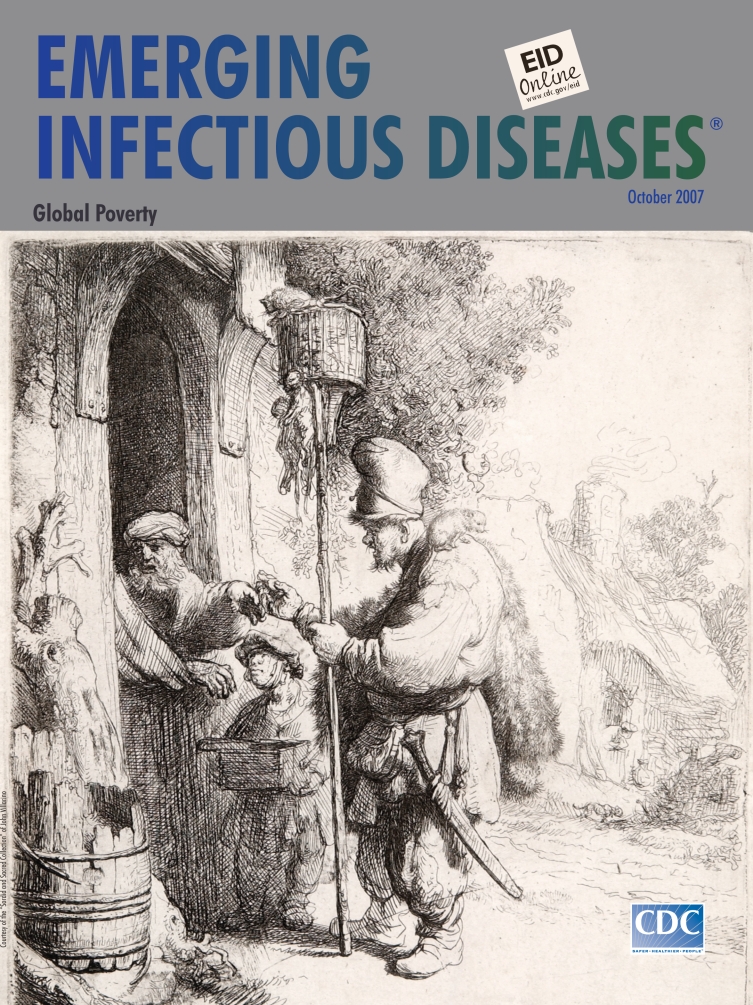
**Rembrandt van Rijn (1606–1669). The Rat Catcher (1632).** Etching (13 cm × 14 cm). Courtesy of the “Sordid and Sacred Collection” of John Villarino

“I frankly consider him a great virtuoso,” said Italian painter Guercino of Rembrandt van Rijn (see also www.cdc.gov/ncidod/EID/vol12no03/about_cover.htm). Guercino (1591–1666) was referring to the legendary master not as painter or portraitist but as etcher because it was etchings that built Rembrandt’s reputation during his lifetime (*1*). The first to fully exploit this technique, which dates back to 14th-century armor ornamentation, he left behind some 300 fine prints, a benchmark for posterity. Most were small, displayed not on walls but in albums like early photographs, rested on tables for a closer look.

Etchings are created by drawing on a resin- or wax-coated metal plate with a needle. The plate is immersed in acid, which “bites” or eats away the lines where the metal is exposed. Rembrandt viewed this technique as drawing. Attracted to its spontaneity, he practiced it throughout his career and defined it with inventiveness and flair. His genius lay in the light touch of the draftsman, not the heavy hand of the professional printmaker. Using the needle as brush or pen, he created lines that flowed across the plates, varied in texture and tone from being carved more deeply or immersed longer in the acid bath (*2*).

For even greater tonal variation, Rembrandt experimented with drypoint—drawing lines directly into the soft surface of the copper plate. These lines held more ink and formed velvety black, rich shadows. Combined etching and drypoint limited copies from a single plate, sometimes to as few as 15, from possible hundreds. His finest prints are rare and unique, even if created by a reproductive process. Unlike other 17th-century artists, who did the drawing, then turned the task over to printmakers, he did the total job himself, able to alter the drawing throughout the process and create, from the same plate, prints that were not identical. The paper used, common European white or thin absorbent, ivory-yellow or light gray Japanese, also produced remarkable variations.

On these small plates, Rembrandt etched life in his native Holland. To capture natural movement, he ran a theatrical studio, where apprentices played out gestures for each other and enacted scenes from the streets of Amsterdam and the fringes of society. Enlivened with dramatic light and shadow, these indelible scenes established his reputation as master storyteller. They described an urban culture conscious of boundaries and criteria for inclusion and exclusion; focused on work, thrift, and restraint; and overwrought with vagabonds, landlopers, beggars, tricksters, outsiders—a population both created and demonized by society and portrayed in sordid detail by period art (*3*).

Earlier painters, Hieronymus Bosch (c.1450–1516) for one, depicted beggars as indistinguishable from demons. Peter Bruegel the Elder (c.1525–1569) painted peasants as objects of mirth, and Adriaen van de Venne (1589–1662) capped his unsparing images of the poor with ironic humor and ridicule. Some of Rembrandt’s prints show the poor in unflattering and compromising situations, but he was indebted to Jacques Callot (c.1592–1635), whose etchings he collected. They humanized the poor and allowed them individuality and seriousness (*4*).

Rembrandt may have learned compassion from his own misfortunes. His life, which might have been one of comfort, recognition, and wealth, turned into a journey of adversity, marred by the deaths of his wife and young children, bankruptcy, and social rejection in his later years. Bitter and disillusioned, he continued to produce earthy street scenes crowded with beggars, peddlers, the underclass, whose faces were oddly reminiscent of his own portraits or those he painted in religious scenes.

In the disorderly intimacy of the streets that so fascinated Rembrandt, economic delinquency manifested itself in more than just the unwanted poor. Stray animals, a hog here and there, tame pigeons, cats, rabid dogs, roamed unchecked. And rats, most prolific, most hated and feared, for as the poet put it, “They fought the dogs and killed the cats, / and bit the babies in the cradles, / and ate the cheeses out of the vats, / And licked the cooks’ own ladles ...” (*5*).

The house rat, or black rat, arrived in Europe around 400 to 200 bce and quickly established a commensal relationship with the locals in homes, ships, river banks, and sewers, generating brisk business for the rat poison peddlers, common street venders. The Pied Piper of Hamelin, a legend immortalized by the Brothers Grimm, recounted rat infestation so severe in that German town, in 1284, it required special intervention.

When Europe was overrun by the Black Death in 1348, Giovanni Boccaccio, who lived through it in the city of Florence, wrote in The Decameron, “It began with yong children, male and female, either under the armepits, or in the groine by certaine swellings, in some to the bignesse of an Apple, in others like an Egge ...” (*6*). His description led to later speculation that the disease was bubonic plague caused by *Yersinia pestis* and spread by fleas carried by the black rat. In A Journal of the Plague Year, Daniel Defoe’s chronicle of the great plague of London in 1665, rats were named as suspects, “All possible Endeavours were used also to destroy the Mice and Rats, especially the latter” (*7*).

In The Rat Catcher, on this month’s cover, Rembrandt’s light hand scratched a telling rat’s tale in a local transaction between an itinerant peddler and a homeowner. Looking on is the peddler’s diminutive assistant, holding a container with rat poison or ferrets trained to hunt rats. The peddler’s extended hand holds poison. The homeowner reaches from behind the half-closed double door but is repulsed. A basket on the long pole is filled with live rats. One on top is poised to jump. Others hang dead from the base. Hairy and unkempt, the peddler himself looks like a rat in his tattered furry cape and long sword. Domestic clutter frames the entrance to the cottage. Etched softly in the background is a prowling cat.

The Rat Catcher was a popular print, copied 11 times in the 17th century (*8*). The scene struck a nerve because it contained much more than a transaction on a village lane. At the threshold of this cottage, separated by the closed half door, met but remained apart, two sections of society: the rooted and the vagrant outsider. What Rembrandt managed to convey was the humanity of all, not just the squeamish owner but the vagrants too, social outcasts though they were. The assistant wears a wistful glance. The peddler, solid on his feet, has a pet perched trustingly on his shoulder.

A carrier of bubonic plague, epidemic typhus, trench fever, ratbite fever, leptospirosis, hantavirus pulmonary syndrome, salmonella poisoning, and many other infections, the rat is still a suspect around the world, destroying as much as one third the global food supply each year, killing domesticated animals, damaging buildings and furnishings (*9*). Social inequity also continues, perpetuating a cycle of poverty and disease (*10*). This cycle cannot be ignored for, as Boccaccio and Defoe reported, disease cannot be fenced out of prosperous areas. “... [T]he plague bacillus never dies ... it bides its time in bedrooms, cellars, trunks, and bookshelves,” wrote Albert Camus, in The Plague, “and perhaps the day would come when, for the bane and the enlightening of men, it would rouse up its rats again and send them forth to die in a happy city” (*11*).
